# 2,2′-Diazinodimethylidyne)di-*o*-phenyl­ene) dibenzoate

**DOI:** 10.1107/S1600536808010155

**Published:** 2008-04-18

**Authors:** Basab Chattopadhyay, Sharmila Basu, Somnath Ghosh, Madeleine Helliwell, Monika Mukherjee

**Affiliations:** a Department of Solid State Physics, Indian Association for the Cultivation of Science, Jadavpur, Kolkata 700 032, India; b Department of Physics, Jadavpur University, Kolkata 700 032, India; c Department of Chemistry, Jadavpur University, Kolkata 700 032, India; d Department of Chemistry, University of Manchester, Manchester M13 9PL, England

## Abstract

The title compound, C_28_H_20_N_2_O_4_, was synthesized by the reaction of 2-(hydrazonometh­yl)phenyl benzoate with iodine. The mol­ecule possesses a crystallographically imposed center of symmetry at the mid-point of the hydrazine N—N bond. The substituents at the ends of the C=N bonds adopt an *E*,*E* configuration. Inter­molecular C—H⋯π(arene) hydrogen bonds and aromatic π–π stacking inter­actions [centroid–centroid distance 3.900 (1) Å] link the mol­ecules into (100) sheets. In addition, there is an inter­molecular C—H⋯O hydrogen-bond inter­action.

## Related literature

For related literature, see: Glaser *et al.* (1995 ▶); Kesslen *et al.* (1999 ▶); Hunig *et al.* (2000 ▶); Glidewell *et al.* (2006 ▶); Xu & Hu (2007 ▶); Zheng *et al.* (2006 ▶); Liu *et al.* (2007 ▶).
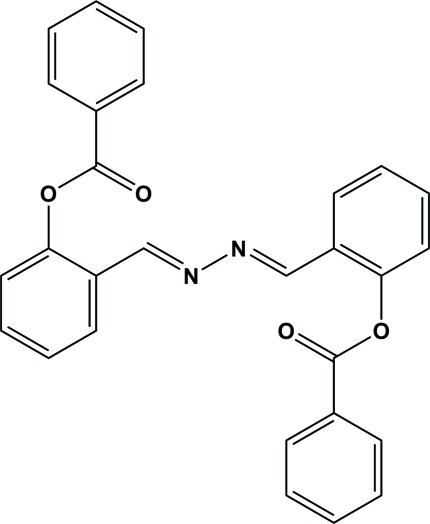


## Experimental

### 

#### Crystal data

C_28_H_20_N_2_O_4_
*M* *_r_* = 448.46
Triclinic, 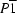

*a* = 5.5442 (9) Å
*b* = 7.9966 (13) Å
*c* = 13.455 (2) Å
α = 73.201 (2)°
β = 82.066 (3)°
γ = 74.441 (2)°
*V* = 548.94 (15) Å^3^
*Z* = 1
Mo *K*α radiation
μ = 0.09 mm^−1^
*T* = 100 (2) K
0.35 × 0.20 × 0.20 mm

#### Data collection

Bruker SMART CCD area-detector diffractometer
Absorption correction: none
2797 measured reflections
1885 independent reflections
1692 reflections with *I* > 2σ(*I*)
*R*_int_ = 0.117

#### Refinement

*R*[*F* ^2^ > 2σ(*F* ^2^)] = 0.051
*wR*(*F* ^2^) = 0.135
*S* = 1.03
1885 reflections
154 parameters
H-atom parameters constrained
Δρ_max_ = 0.24 e Å^−3^
Δρ_min_ = −0.28 e Å^−3^

### 

Data collection: *SMART* (Bruker, 2007 ▶); cell refinement: *SAINT* (Bruker, 2007 ▶); data reduction: *SAINT*; program(s) used to solve structure: *SHELXS97* (Sheldrick, 2008 ▶); program(s) used to refine structure: *SHELXL97* (Sheldrick, 2008 ▶); molecular graphics: *DIAMOND* (Brandenburg, 1999 ▶); software used to prepare material for publication: *SHELXL97* and *PLATON* (Spek, 2003 ▶).

## Supplementary Material

Crystal structure: contains datablocks I, global. DOI: 10.1107/S1600536808010155/fj2111sup1.cif

Structure factors: contains datablocks I. DOI: 10.1107/S1600536808010155/fj2111Isup2.hkl

Additional supplementary materials:  crystallographic information; 3D view; checkCIF report

## Figures and Tables

**Table 1:** Hydrogen-bond geometry (Å, °)

*D*—H⋯*A*	*D*—H	H⋯*A*	*D*⋯*A*	*D*—H⋯*A*
C6—H6⋯O2^i^	0.95	2.64	3.519 (2)	154
C5—H5⋯*Cg*1^ii^	0.95	2.79	3.510 (2)	133

Symmetry codes: (i) 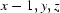; (ii) 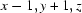. *Cg*1 is the centroid of the C9–C14 ring.

**Fig. 1.**
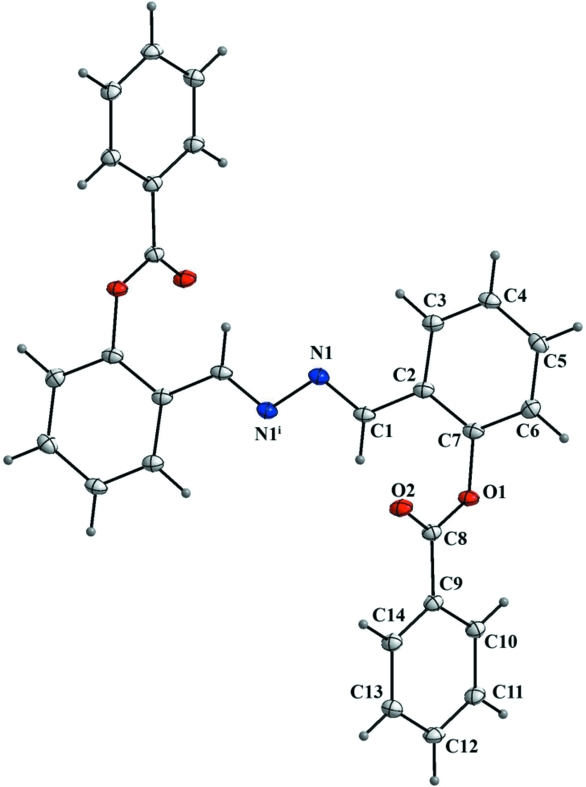
View of the molecule of (I), with displacement ellipsoids drawn at the 30% probability level.[Symmetry code (i):- -x, -y, -z]

**Fig. 2.**
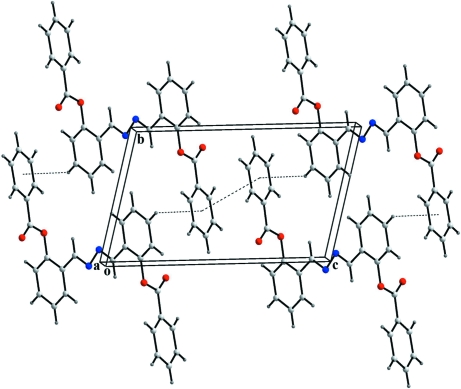
The packing of (I), viewed along the a axis, showing intermolecular C—H···π (arene) hydrogen bond and aromatic π···π stacking interaction.

## supplementary crystallographic information

### Comment

The synthetic utility of hydrazine compounds in coordination chemistry as well as their remarkable photochromic properties has resulted in continued interest in studies of their stereochemistry (Glaser *et al.*, 1995). The photochromism in hydrazines arises from intramolecular H-atom transfer, together with a change in the π-electron system. To study the effect of intermolecular interactions, such as π···π charge transfer or hydrogen bonding, on H-atom transfer processes, solid state structure analyses of a number of hydrazine compounds containing both a diamine linkage and N—N bonding have been reported in the literature (Liu *et al.*, 2007; Xu & Hu, 2007; Zheng *et al.*, 2006) We report here the synthesis and molecular structure of the title benzylidenehydrazine derivative(I).

As observed in many symmetric azines with an E, E configuration (Glidewell *et al.*, 2006), the molecule of (I) possesses a crystallographically imposed center of symmetry at the mid-point of the N—N bond (Fig. 1). Consequently the asymmetric unit consists of half of the molecule. The central –CH=N—N=CH– fragment is strictly planar, but as a whole the molecule is not planar; the benzoyloxy group (C8—C14, O1, O2) is rotated about the O1—C7 bond by 78.7 (2)° with respect to the plane of the benzylidene hydrazine moiety (C1—C7, N1). The single-bond character of N1—N1^i^[1.408 (2) Å] and the double-bond character of C1=N1[1.274 (2) Å] indicate a lack of delocalization of π-electrons, while the planar structure of >C=N—N=C< chain indicates π configuration. The C=N—N angle [11.4 (2)°] in (I) is significantly smaller than the ideal *sp*^2^value of 120°, as consequence of repulsion between the nitrogen lone pairs and the adjacent C=N bond.

The supramolecular aggregation in (I) is determined by C—H···π (arene) hydrogen bond and aromatic π···π stacking interactions. The aryl C5 atom in the ring at (*x*, *y*, *z*) is part of the molecule centered across (0, 0, 0) and acts as a hydrogen bond donor to the aryl ring (C9—C14) at (-1 + *x*, 1 + *y*, *z*), which forms part of the molecule centered across (-1, 1, 0). Propagation of this hydrogen-bond forms a chain running parallel to the [11 0] direction (Fig. 2). The phenyl rings (C9—C14) at (*x*, *y*, *z*) and (1 - *x*, 1 - *y*,1 - *z*) are components of the molecules across the inversion centers at (0, 0, 0) and (1 - *x*, 1 - *y*, 1 - *z*), respectively. These strictly parallel rings with an interplanar spacing of 3.464 (1) Å, the ring-centroid separation of 3.900 (1)Å and the centroid offset of 1.79Å lead to the formation of a π-stacked chain of centrosymmetric molecules running parallel to the [1 1 1] direction (Fig. 2).The combination of the [110] and [1 1 1] chains generates a (100) sheet.

### Experimental

A solution of iodine(8 g, 7 mmol) in 15 ml tetrahydrofuran (THF) was added dropwise to a magnetically stirred solution of 2- benzoyloxy phenyl hydrazone (0.68 g, 2.8 mmol) in THF (40 ml) and triethylamine (10 ml) at room temperature (298k). The mixture was stirred for 1 h and then diluted with water (100 ml) and extracted with ether (3x30 ml). The extract was washed with water, aqueous sodium thiosulfate solution and brine followed by drying over anhydrous sodium sulfate. The solvent was removed *in vacuo*. The residual black oil was dissolved in carbon tetrachloride and filtered through silica gel to give a light yellow oil which on standing yielded shinny yellow crystals of the title compound (I).

### Refinement

All H atoms were positioned geometrically and refined using a riding model with *U*_iso_(H) values fixed at 1.2Ueq(C).

### Crystal data

**Table:**

C_28_H_20_N_2_O_4_	*Z* = 1
*M**_r_* = 448.46	*F*_000_ = 234
Triclinic, *P*1	*D*_x_ = 1.357 Mg m^−^^3^
Hall symbol: -P 1	Mo *K*α radiation λ = 0.71073 Å
*a* = 5.5442 (9) Å	Cell parameters from 1976 reflections
*b* = 7.9966 (13) Å	θ = 2.4–27.5º
*c* = 13.455 (2) Å	µ = 0.09 mm^−^^1^
α = 73.201 (2)º	*T* = 100 (2) K
β = 82.066 (3)º	Block, pale yellow
γ = 74.441 (2)º	0.35 × 0.20 × 0.20 mm
*V* = 548.94 (15) Å^3^	

### Data collection

**Table:**

Bruker SMART CCD area-detector diffractometer	1692 reflections with *I* > 2σ(*I*)
Radiation source: fine-focus sealed tube	*R*_int_ = 0.117
Monochromator: graphite	θ_max_ = 25.0º
*T* = 100(2) K	θ_min_ = 1.6º
φ and ω scans	*h* = −6→3
Absorption correction: none	*k* = −9→9
2797 measured reflections	*l* = −15→15
1885 independent reflections	

### Refinement

**Table:**

Refinement on *F*^2^	Secondary atom site location: difference Fourier map
Least-squares matrix: full	Hydrogen site location: inferred from neighbouring sites
*R*[*F*^2^ > 2σ(*F*^2^)] = 0.051	H-atom parameters constrained
*wR*(*F*^2^) = 0.135	*w* = 1/[σ^2^(*F*_o_^2^) + (0.0874*P*)^2^ + 0.0721*P*] where *P* = (*F*_o_^2^ + 2*F*_c_^2^)/3
*S* = 1.03	(Δ/σ)_max_ < 0.001
1885 reflections	Δρ_max_ = 0.24 e Å^−^^3^
154 parameters	Δρ_min_ = −0.28 e Å^−^^3^
Primary atom site location: structure-invariant direct methods	Extinction correction: none

### Special details

**Table:**

Geometry. All e.s.d.'s (except the e.s.d. in the dihedral angle between two l.s. planes) are estimated using the full covariance matrix. The cell e.s.d.'s are taken into account individually in the estimation of e.s.d.'s in distances, angles and torsion angles; correlations between e.s.d.'s in cell parameters are only used when they are defined by crystal symmetry. An approximate (isotropic) treatment of cell e.s.d.'s is used for estimating e.s.d.'s involving l.s. planes.
Refinement. Refinement of *F*^2^ against ALL reflections. The weighted *R*-factor *wR* and goodness of fit *S* are based on *F*^2^, conventional *R*-factors *R* are based on *F*, with *F* set to zero for negative *F*^2^. The threshold expression of *F*^2^ > σ(*F*^2^) is used only for calculating *R*-factors(gt) *etc*. and is not relevant to the choice of reflections for refinement. *R*-factors based on *F*^2^ are statistically about twice as large as those based on *F*, and *R*- factors based on ALL data will be even larger.

### Fractional atomic coordinates and isotropic or equivalent isotropic displacement parameters (Å^2^)

**Table:**

	*x*	*y*	*z*	*U*_iso_*/*U*_eq_	
O1	−0.60203 (19)	−0.17401 (13)	0.23034 (8)	0.0250 (3)	
O2	−0.3270 (2)	−0.15072 (14)	0.33163 (8)	0.0299 (3)	
N1	−0.1081 (2)	0.06358 (16)	0.01102 (9)	0.0256 (3)	
C1	−0.2578 (3)	−0.01150 (19)	0.07886 (11)	0.0246 (4)	
H1	−0.2140	−0.1383	0.1073	0.029*	
C2	−0.4964 (3)	0.09474 (19)	0.11375 (11)	0.0239 (4)	
C3	−0.5716 (3)	0.2813 (2)	0.07164 (12)	0.0273 (4)	
H3	−0.4643	0.3410	0.0206	0.033*	
C4	−0.7999 (3)	0.3799 (2)	0.10330 (12)	0.0291 (4)	
H4	−0.8471	0.5066	0.0747	0.035*	
C5	−0.9593 (3)	0.2945 (2)	0.17645 (12)	0.0287 (4)	
H5	−1.1170	0.3624	0.1972	0.034*	
C6	−0.8898 (3)	0.1100 (2)	0.21961 (11)	0.0263 (4)	
H6	−0.9986	0.0508	0.2700	0.032*	
C7	−0.6600 (3)	0.01385 (19)	0.18816 (11)	0.0240 (4)	
C8	−0.4341 (3)	−0.24172 (19)	0.30508 (11)	0.0228 (4)	
C9	−0.3993 (3)	−0.43981 (19)	0.34806 (11)	0.0231 (4)	
C10	−0.2123 (3)	−0.5285 (2)	0.41615 (12)	0.0264 (4)	
H10	−0.1100	−0.4633	0.4326	0.032*	
C11	−0.1736 (3)	−0.7120 (2)	0.46053 (12)	0.0283 (4)	
H11	−0.0454	−0.7729	0.5076	0.034*	
C12	−0.3236 (3)	−0.8067 (2)	0.43577 (12)	0.0293 (4)	
H12	−0.2985	−0.9326	0.4664	0.035*	
C13	−0.5087 (3)	−0.7186 (2)	0.36691 (12)	0.0287 (4)	
H13	−0.6087	−0.7845	0.3496	0.034*	
C14	−0.5495 (3)	−0.5342 (2)	0.32285 (12)	0.0264 (4)	
H14	−0.6781	−0.4733	0.2761	0.032*	

### Atomic displacement parameters (Å^2^)

**Table:**

	*U*^11^	*U*^22^	*U*^33^	*U*^12^	*U*^13^	*U*^23^
O1	0.0317 (6)	0.0174 (6)	0.0274 (6)	−0.0070 (4)	−0.0069 (4)	−0.0048 (4)
O2	0.0394 (6)	0.0212 (6)	0.0337 (6)	−0.0127 (5)	−0.0095 (5)	−0.0061 (4)
N1	0.0305 (7)	0.0198 (6)	0.0267 (7)	−0.0039 (5)	−0.0036 (5)	−0.0077 (5)
C1	0.0334 (8)	0.0167 (7)	0.0249 (7)	−0.0057 (6)	−0.0065 (6)	−0.0058 (6)
C2	0.0307 (8)	0.0199 (8)	0.0233 (7)	−0.0067 (6)	−0.0049 (6)	−0.0073 (6)
C3	0.0328 (8)	0.0211 (8)	0.0280 (8)	−0.0075 (6)	−0.0037 (6)	−0.0049 (6)
C4	0.0362 (9)	0.0177 (7)	0.0329 (8)	−0.0037 (6)	−0.0076 (6)	−0.0061 (6)
C5	0.0290 (8)	0.0261 (8)	0.0326 (8)	−0.0023 (6)	−0.0052 (6)	−0.0129 (6)
C6	0.0294 (8)	0.0265 (8)	0.0264 (8)	−0.0095 (6)	−0.0026 (6)	−0.0093 (6)
C7	0.0317 (8)	0.0176 (7)	0.0257 (8)	−0.0062 (6)	−0.0089 (6)	−0.0068 (6)
C8	0.0270 (7)	0.0201 (8)	0.0224 (7)	−0.0067 (6)	−0.0005 (6)	−0.0070 (6)
C9	0.0274 (8)	0.0201 (8)	0.0240 (7)	−0.0077 (6)	0.0005 (6)	−0.0083 (6)
C10	0.0311 (8)	0.0226 (8)	0.0285 (8)	−0.0090 (6)	−0.0039 (6)	−0.0083 (6)
C11	0.0316 (8)	0.0223 (8)	0.0293 (8)	−0.0043 (6)	−0.0047 (6)	−0.0050 (6)
C12	0.0360 (9)	0.0169 (7)	0.0337 (8)	−0.0064 (6)	0.0019 (6)	−0.0065 (6)
C13	0.0318 (8)	0.0222 (8)	0.0366 (9)	−0.0108 (6)	−0.0003 (6)	−0.0114 (6)
C14	0.0283 (8)	0.0214 (8)	0.0310 (8)	−0.0069 (6)	−0.0028 (6)	−0.0081 (6)

### Geometric parameters (Å, °)

**Table:**

O1—C8	1.3580 (18)	C6—C7	1.381 (2)
O1—C7	1.4073 (17)	C6—H6	0.9500
O2—C8	1.2045 (17)	C8—C9	1.489 (2)
N1—C1	1.274 (2)	C9—C10	1.382 (2)
N1—N1^i^	1.408 (2)	C9—C14	1.393 (2)
C1—C2	1.466 (2)	C10—C11	1.384 (2)
C1—H1	0.9500	C10—H10	0.9500
C2—C7	1.390 (2)	C11—C12	1.392 (2)
C2—C3	1.401 (2)	C11—H11	0.9500
C3—C4	1.384 (2)	C12—C13	1.380 (2)
C3—H3	0.9500	C12—H12	0.9500
C4—C5	1.383 (2)	C13—C14	1.389 (2)
C4—H4	0.9500	C13—H13	0.9500
C5—C6	1.387 (2)	C14—H14	0.9500
C5—H5	0.9500		
			
C8—O1—C7	116.52 (10)	C2—C7—O1	120.43 (13)
C1—N1—N1^i^	111.37 (15)	O2—C8—O1	123.28 (13)
N1—C1—C2	121.02 (13)	O2—C8—C9	125.08 (13)
N1—C1—H1	119.5	O1—C8—C9	111.63 (12)
C2—C1—H1	119.5	C10—C9—C14	120.38 (14)
C7—C2—C3	117.32 (14)	C10—C9—C8	117.36 (13)
C7—C2—C1	121.33 (13)	C14—C9—C8	122.25 (13)
C3—C2—C1	121.33 (13)	C9—C10—C11	120.26 (13)
C4—C3—C2	120.94 (14)	C9—C10—H10	119.9
C4—C3—H3	119.5	C11—C10—H10	119.9
C2—C3—H3	119.5	C10—C11—C12	119.53 (14)
C5—C4—C3	120.14 (14)	C10—C11—H11	120.2
C5—C4—H4	119.9	C12—C11—H11	120.2
C3—C4—H4	119.9	C13—C12—C11	120.28 (14)
C4—C5—C6	120.20 (14)	C13—C12—H12	119.9
C4—C5—H5	119.9	C11—C12—H12	119.9
C6—C5—H5	119.9	C12—C13—C14	120.35 (13)
C7—C6—C5	118.95 (14)	C12—C13—H13	119.8
C7—C6—H6	120.5	C14—C13—H13	119.8
C5—C6—H6	120.5	C13—C14—C9	119.19 (14)
C6—C7—C2	122.43 (14)	C13—C14—H14	120.4
C6—C7—O1	117.04 (13)	C9—C14—H14	120.4
			
N1^i^—N1—C1—C2	−179.77 (13)	C8—O1—C7—C2	78.69 (16)
N1—C1—C2—C7	−179.63 (13)	C7—O1—C8—O2	−3.9 (2)
N1—C1—C2—C3	1.9 (2)	C7—O1—C8—C9	176.64 (11)
C7—C2—C3—C4	0.0 (2)	O2—C8—C9—C10	−6.7 (2)
C1—C2—C3—C4	178.48 (13)	O1—C8—C9—C10	172.76 (12)
C2—C3—C4—C5	−0.9 (2)	O2—C8—C9—C14	172.44 (14)
C3—C4—C5—C6	1.0 (2)	O1—C8—C9—C14	−8.13 (19)
C4—C5—C6—C7	−0.1 (2)	C14—C9—C10—C11	−0.4 (2)
C5—C6—C7—C2	−0.8 (2)	C8—C9—C10—C11	178.68 (12)
C5—C6—C7—O1	−177.14 (12)	C9—C10—C11—C12	0.3 (2)
C3—C2—C7—C6	0.9 (2)	C10—C11—C12—C13	0.4 (2)
C1—C2—C7—C6	−177.63 (13)	C11—C12—C13—C14	−1.0 (2)
C3—C2—C7—O1	177.10 (12)	C12—C13—C14—C9	0.8 (2)
C1—C2—C7—O1	−1.4 (2)	C10—C9—C14—C13	−0.1 (2)
C8—O1—C7—C6	−104.90 (14)	C8—C9—C14—C13	−179.14 (12)

Symmetry codes: (i) −*x*, −*y*, −*z*.

### Hydrogen-bond geometry (Å, °)

**Table:**

*D*—H···*A*	*D*—H	H···*A*	*D*···*A*	*D*—H···*A*
C6—H6···O2^ii^	0.95	2.64	3.519 (2)	154
C5—H5···Cg1^iii^	0.95	2.79	3.510 (2)	133

Symmetry codes: (ii) *x*−1, *y*, *z*; (iii) *x*−1, *y*+1, *z*.
